# IL-6 is Upregulated in Late-Stage Disease in Monkeys Experimentally Infected with *Trypanosoma brucei rhodesiense*

**DOI:** 10.1155/2013/320509

**Published:** 2013-09-30

**Authors:** Dawn Nyawira Maranga, John Maina Kagira, Christopher Kariuki Kinyanjui, Simon Muturi Karanja, Naomi Wangari Maina, Maina Ngotho

**Affiliations:** ^1^Jomo Kenyatta University of Agriculture and Technology, College of Health Sciences, Biochemistry Department, P.O. Box 62000-00200, Nairobi, Kenya; ^2^Institute of Primate Research, Animal Science Department, P.O. Box 24481-00502, Nairobi, Kenya; ^3^Jomo Kenyatta University of Agriculture and Technology, College of Agriculture and Natural Resources, Animal Health and Production Department, P.O. Box 62000-00200, Nairobi, Kenya

## Abstract

The management of human African trypanosomiasis (HAT) is constrained by lack of simple-to-use diagnostic, staging, and treatment tools. The search for novel biomarkers is, therefore, essential in the fight against HAT. The current study aimed at investigating the potential of IL-6 as an adjunct parameter for HAT stage determination in vervet monkey model. Four adult vervet monkeys (*Chlorocebus aethiops*) were experimentally infected with *Trypanosoma brucei rhodesiense* and treated subcuratively at 28 days after infection (dpi) to induce late stage disease. Three noninfected monkeys formed the control group. Cerebrospinal fluid (CSF) and blood samples were obtained at weekly intervals and assessed for various biological parameters. A typical HAT-like infection was observed. The late stage was characterized by significant (*P* < 0.05) elevation of CSF IL-6, white blood cell count, and total protein starting 35 dpi with peak levels of these parameters coinciding with relapse parasitaemia. Brain immunohistochemical staining revealed an increase in brain glial fibrillary acidic protein expression indicative of reactive astrogliosis in infected animals which were euthanized in late-stage disease. The elevation of IL-6 in CSF which accompanied other HAT biomarkers indicates onset of parasite neuroinvasion and show potential for use as an adjunct late-stage disease biomarker in the Rhodesian sleeping sickness.

## 1. Introduction

Human African trypanosomiasis (HAT) is a tropical infectious disease caused by the protozoan parasites *Trypanosoma brucei rhodesiense* and *T. b. gambiense*. *T. b. rhodesiense* causes an acute illness in eastern Africa, while *T. b. gambiense* causes a chronic disease in western and central Africa. The disease is classified as a neglected disease of poverty with 60 million people at risk and only 5 million under active surveillance or with health centre access [1, 2]. In Kenya, recent cases of *T. b. rhodesiense* sleeping sickness have been reported in tourists visiting the Maasai Mara Game Reserve [3, 4] emphasizing the need for efficient disease surveillance and control. Currently, the management of human African trypanosomiasis (HAT) is mainly constrained by lack of simple-to-use diagnostic, staging, and treatment tools. The current criteria used in disease staging is primarily based on the detection of trypanosomes in CSF and/or WCC > 5 cells/*µ*L and/or total protein > 37 mg/100 mL in CSF [5], the validity of this criterion is, however, debated, and newer, more specific markers are being developed [6–8].

After the infective tsetse fly bite, trypanosomes initially proliferate in the blood and lymphatic system characterizing the early stage. As the disease progresses, the trypanosomes invade the central nervous system (CNS) leading to the late-stage [9]. However, the earliest time of blood-brain-barrier (BBB) penetration remains unknown. Late-stage disease is characterized by parasite invasion of meninges and choroid plexus with IL-6, known to be involved in BBB modulation, also playing a role in the accompanied activation of astrocytes in the brain and eventual inflammation of the brain (meningoencephalitis) [10, 11].

In late-stage *T. b. rhodesiense* human infections, abnormally high CSF IL-6 and IL-10 were observed, decreasing only after treatment indicative of potential for use in staging and treatment monitoring. Additionally, mouse model studies have also shown significant increases in brain IL-6 expression that correlated with astrocyte activation [12]. Vervet monkeys have been shown to develop a disease clinically and immunologically similar to that in humans [13, 14] with three model disease stages described [13]; early (0–14 dpi), transitional (21–28), and advanced late-stage (35–61 dpi). Recent vervet studies have shown immunological responses paralleling the onset of CNS disease with peak levels coinciding with meningoencephalitis [14] and astrocyte activation [15].

The monkeys, unlike rodents, allow for sequential collection of CSF enabling study of changes in the CNS. A more rapid late-stage laboratory animal model for HAT was recently described [14]. The current study investigated the profile of CSF IL-6, total protein, total white cell changes, and activation of astrocytes in the lead up to pathological lesions indicative of meningoencephalitis in this monkey model.

## 2. Materials and Methods

### 2.1. *Trypanosomes*

*Trypanosoma brucei rhodesiense* isolate IPR 001 was used in this study. It was isolated from the cerebrospinal fluid of a late-stage HAT patient in Bugiri, Uganda, in 2008 [14]. The isolate was passaged thrice in irradiated (500 Rad) Swiss white mice before cryopreservation in liquid nitrogen.

### 2.2. Experimental Animals

Seven vervet monkeys of both sexes, weighing 2.0–6.0 kg, with males weighing between 4.0-5.0 kg, were recruited for the study. The animals underwent a 90-day quarantine, during which they were screened for zoonotic diseases and treated for ecto- and endoparasites before being subjected to the experiment. They were trained for ease of adaptation and maintained on commercial chow (Goldstar Feeds Ltd., Nairobi, Kenya) supplemented with fresh fruits and vegetables. Drinking water was provided *ad libitum*. The monkeys were housed in stainless steel cages at ambient room temperatures of 18–25°C, under biosafety level II animal holding conditions and were allowed visual contact with conspecifics.

### 2.3. Study Design

Four monkeys were infected intravenously with approximately 10^4^ trypanosomes, delivered in 1 mL of phosphate saline glucose, while the noninfected control consisted of three monkeys. The infected animals were given subcurative treatment with diminazene aceturate (DA) (Veriben, Sanofi, Paris, France) at 5 mg/kg body weight (bwt) via intramuscular injection for three consecutive days, starting 28 days after infection (28 dpi). A daily clinical evaluation of the appetite, clinical appearance, and disease symptoms was conducted during the study. The parasitaemia was scored daily using the method as previously described [16]. Upon relapse of trypanosomes, the infected animals were euthanized, and postmortem examination was undertaken.

### 2.4. Sample Collection and Initial Analysis

The monkeys were anesthetized weekly using ketamine hydrochloride (Agrar Agrar Holland BV, Soest, the Netherlands) at dosage of 10 mg/kg bwt. One mL of CSF was obtained via lumbar puncture and examined for presence of trypanosomes and white blood cells as previous described [14, 15]. Two mL of blood for serum separation was also drawn from the femoral vein. Upon euthanasia at the end of the study, brain tissue from infected animals was harvested and preserved in 10% formalin.

### 2.5. IL-6 Cytokine Cytometric Assay

The non-human primate Th1/Th2 Cytokine Cytometry Bead Array (CBA) kit and the BD CBA software (BD Biosciences, USA) were used to quantitatively measure IL-6 levels as previously described [17]. After the acquisition of sample data using a flow cytometer (BD Facscalibur, Santa Ana, USA), the sample results were described in graphical and tabular format using the BD CBA analysis software (BD Biosciences, USA).

### 2.6. Total Protein Assay

Total protein was analysed in the CSF using the modified Coomasie Brilliant Blue Total Protein test kit (BIORAD, USA) as previously described [18]. 

### 2.7. Brain Immunohistochemistry

The harvested brain tissues from infected animals were trimmed and processed as previously described [14, 15]. Wax-mounted histological sections were then cut on a microtome to 5 *µ*m thickness, mounted onto slides, and stained with glial fibrillary acetic protein (GFAP) as previously described [15].

### 2.8. Data Analysis

Data are presented as line graphs depicting means ± SEM. Differences between means were compared using the Student's *t*-test and ANOVA. The differences in means were considered statistically significant when *P* < 0.05.

### 2.9. Ethical Review

All protocols and procedures used in the current study were reviewed and approved by the Institutional Review Committee (IRC) of the Institute of Primate Research (IPR), Kenya.

## 3. Results

### 3.1. Clinical Signs and Parasitaemia

The early stage clinical signs in the infected animals included: fever, dullness, enlarged lymph nodes and spleen, weight loss, increased respiratory and pulse rates, and peri-orbital erythema. Late-stage clinical signs included increased aggression, hind-leg paresis and paralysis, and sleepiness, which were observed between 42 and 56 dpi at which points the animals were euthanised. One animal developed a fulminant disease and was euthanised at 9 dpi. The necropsy features of this monkey showed extensive petechiation of serosal membranes, grossly enlarged heart, liver, spleen, and other organs indicating development of an acute fulminant disease. In the remaining monkeys, treatment with DA at 28 dpi cleared trypanosomes in blood and relapse occurred in various animals between 49 and 56 dpi. 

### 3.2. CSF Parasitosis and White Cell Count

**Figure 1:**
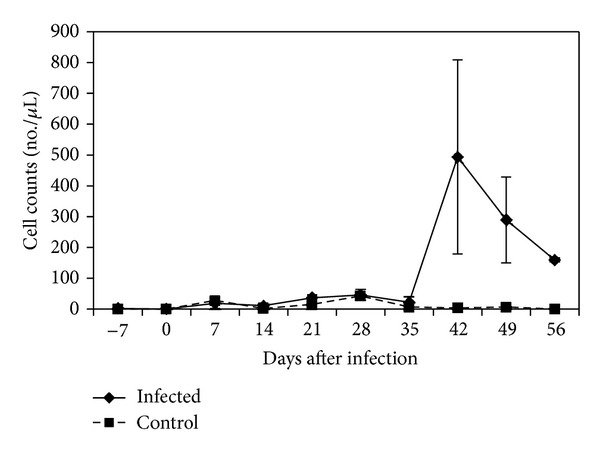
Mean cerebrospinal fluid white cell count in control and *T. b. rhodesiense *IPR 001 infected vervet monkeys. Monkeys were treated with diminazene aceturate at 28 dpi.

The trypanosomes were detected in CSF on 14 dpi. Treatment with DA 28 dpi resulted in parasite clearance in blood and CSF. Parasites reappeared in CSF by 42 dpi with an average of 50 trypanosomes/*µ*L observed. The mean CSF white cell count prior to infection was 1 (range 0–5) cells/*μ*L. This count increased steadily starting 35 dpi to a peak of 494 (range 10–1085) cells/*μ*L (*P* < 0.05), which occurred 42 dpi (Figure 1). There were no changes in CSF white cell counts in uninfected control vervet monkeys during the entire experimental period.

### 3.3. Interleukin-6 (IL-6)

**Figure 2:**
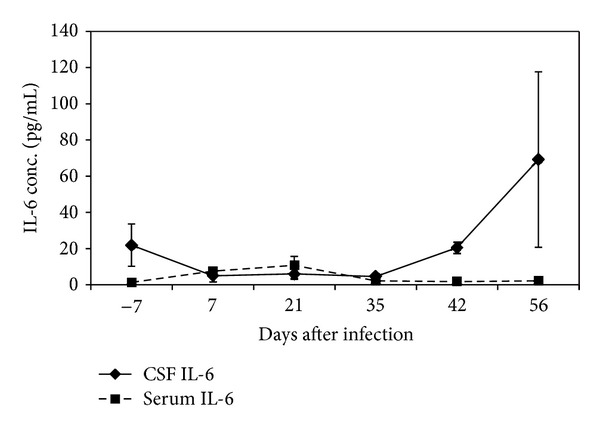
Mean CSF and serum IL-6 levels in *T. b. rhodesiense *IPR 001 infected vervet monkeys. Monkeys were treated with diminazene aceturate at 28 dpi.

Prior to infection, the mean serum concentration of IL-6 in all monkeys was 1.37 pg/mL (range: 1.12–1.43 pg/mL). Upon infection, the serum IL-6 levels increased gradually and were significantly elevated above preinfection levels (*P* < 0.05) with highest levels of 13.7 pg/mL attained on 21 dpi. By 35 dpi, IL-6 levels had decreased to preinfection levels and within range of uninfected control animals. A slight elevation in control animals at 56 dpi was observed although levels remained within range of previous time points. The CSF IL-6 concentrations of infected vervet monkeys remained low within preinfection level range of 1.77–2.34 pg/mL up until 35 dpi. A steady rise resulting in a significant increase of IL-6 (*P* < 0.05) over preinfection levels was observed in the subsequent weeks with the highest level of 69.22 pg/mL recorded on 56 dpi (Figure 2).

### 3.4. Cerebrospinal Fluid Total Protein (TP)

**Figure 3:**
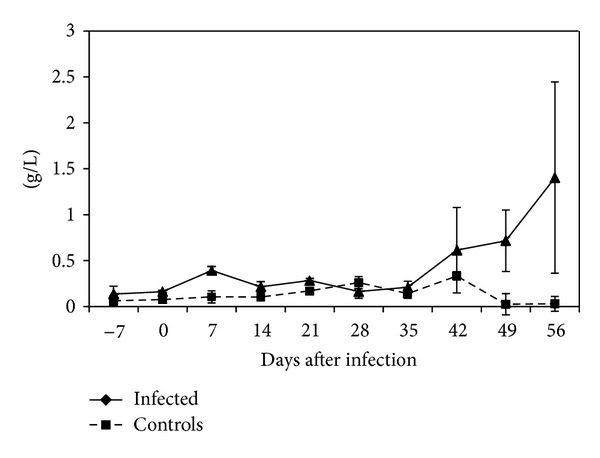
Mean CSF total protein levels in control and *T. b. rhodesiense *IPR 001 infected vervet monkeys. Monkeys were treated with diminazene aceturate at 28 dpi.

A slight peak in CSF protein was observed at 7 dpi with a mean value of 0.47 g/L (range: 0.17–0.74) g/L. Significant elevation above preinfection levels (*P* < 0.05) and normal CSF TP range of 0.11–0.5 g/L was observed starting 35 dpi with highest levels of 1.4033 (Figure 3).

### 3.5. Brain Immunohistochemistry

**Figure 4:**
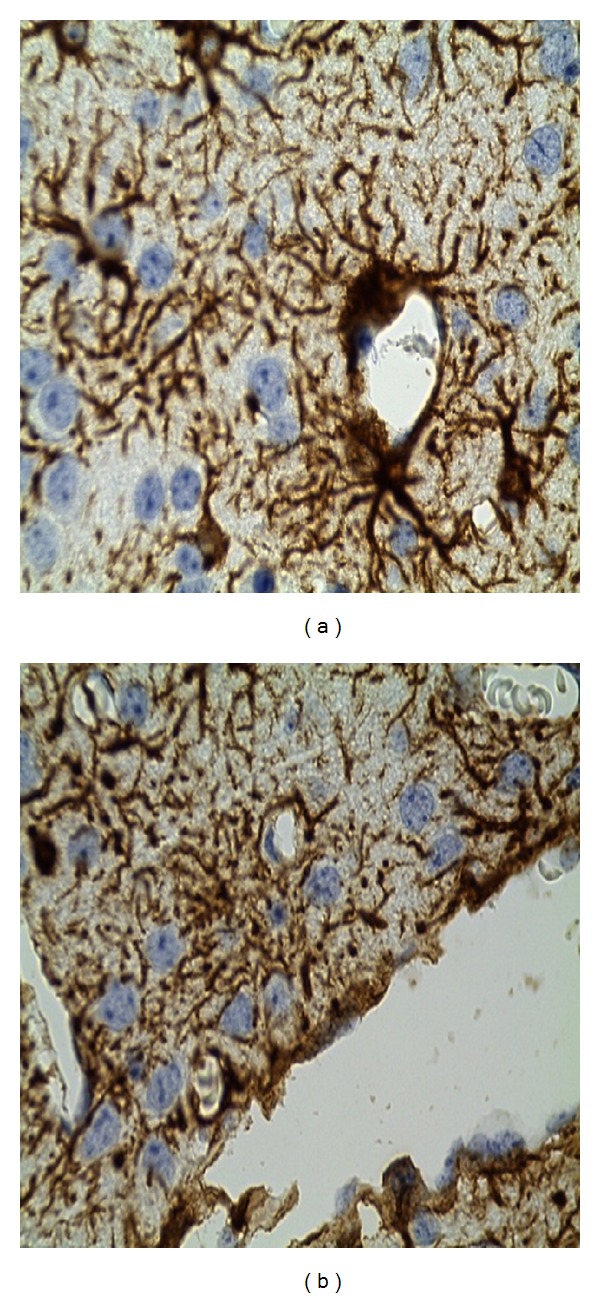
(a) Immunohistological changes in monkey infected with trypanosomiasis and euthanized in late-stage disease. (a) Reactive astrogliosis in (magnification ×100), (b) loss of ependymal lining around brain ventricles (magnification ×100).

The brain specimen of the vervet (vervet 1) which was euthanized on 9 dpi showed astrocytes tiling the entire CNS in a contiguous and essentially nonoverlapping manner that was orderly and well organized. The territories of astrocyte processes did not overlap and many astrocytes did not express GFAP. The brain tissues of monkeys euthanized in late-stage (vervet 2 at 42 dpi, vervets 3 & 4 at 56 dpi) displayed diffuse reactive astrogliosis characterized by pronounced upregulation of GFAP expression, astrocyte hypertrophy, astrocyte proliferation, and pronounced overlap of astrocyte processes resulting in disruption of individual astrocyte domains (Figure 4(a)). There was also loss of ependymal lining around brain ventricles suggesting BBB disruption and meningoencephalitis (Figure 4(b)).

## 4. Discussion

The current study showed that experimental infection of the vervet monkeys with *T. b. rhodesiense* IPR 001 resulted in a typical HAT-like infection characterized by fever, dramatic muscle wasting, weight loss, enlarged lymph nodes, and eventually insomnia, somnolence, and aggressiveness. Additionally, necropsy of the infected monkey euthanized at 9 dpi revealed extensive petechiation of serosal membranes, grossly enlarged heart, liver, spleen, and other organs indicating development of an acute fulminant disease.

In the vervet monkey model as described by Schmidt and Sayer [13], late-stage is assumed to be achieved when relapse parasitaemia occurs. Subsequent vervet monkey models such as the *T. b. rhodesiense* KETRI 2537 model have shown meningoencephalitis (98 dpi) to occur before relapse parasitaemia (112 dpi) [19, 20]. Studies using IPR 001 model indicate acute infection with *T. b. rhodesiense* IPR 001 can result in meningoencephalitis much sooner showing that the timing of late-stage is dependent on the trypanosome isolate [14].

In this model, treatment with DA 28 dpi did not clear the CSF trypanosomes, and this could have been responsible for the shortened period to relapse. In spite of the reduction in time of CNS parasitization in this model, late-stage signs as well as brain lesions were distinct and uniformly observed in the vervet monkeys starting around 42 dpi. Inflammation-related brain pathology has been known to be associated with elevated cytokine levels such as IL-6, TNF-*α*, and IL-10 [12]. Similarly, late-stage HAT in humans is characterized by increase in IL-6 and other inflammatory cytokines [8, 21–23]. In our study, a significant increase in CSF IL-6 concentrations was observed to start 35 dpi coinciding with a marked increase in white blood cell count in the CSF accompanied by astrocytosis and total protein increase. A similar finding was also reported in HAT patients, where significantly elevated CSF IL-6 concentrations were detected in those characterized as being in late-stage, with WCC of >20 cells/*µ*L [8, 23].

Activation of astrocytes in HAT has been linked to trypanosome neuroinvasion in brain with astrocytes acting as mediators of the inflammation process through antigen presentation and the secretion of immunomodulatory cytokines such as IL-6 [24, 25]. Interactions between astrocytes and brain endothelial cells have been shown to affect blood brain barrier (BBB) permeability mediated by astrocyte-released modulating factors [26, 27]. Studies have shown that IL-6 may also play a role in increasing BBB permeability through direct action on endothelial cells leading to increased vascular permeability [28]. Astrocyte activation and the upregulation of IL-6 points towards a more refined timing of late-stage disease in the HAT vervet monkey model. In the current study, marked reactive astrogliosis was observed from 42 dpi coinciding with significant pleocytosis and hyperproteinemia as well as IL-6 increase in the CSF. Elevation of CSF white blood cells and total protein, known indicators of CNS disease [18], and IL-6 is noted from 35 dpi indicative of parasite neuroinvasion and early phases of the meninigoencephalitic stage of the disease. Findings from *T. b. brucei*-infected mice studies are in agreement with these results where a correlation between IL-6 presence in the brain with meningoencephalitis as well as astrocyte activation was observed [11, 12]. The gradual rise of CSF total protein with highest levels being observed at 56 dpi may be explained by blood CSF-barrier (BSFB) dysfunction, observed to occur in the course of CNS disease [18].

Sleeping sickness is known to be characterized by a profound immune dysregulation where molecules involved in the elicited immune response are being investigated for utility as staging markers. A recent study showing IgM, MMP-9, and CXCL13, alone or combined with CXCL10 as promising stage determination markers of *T. b. rhodesiense*-infected patients [29], highlights the potential usefulness of such markers in HAT. IL-6 may prove useful as a staging marker and probably more so in combination with panel of biomarkers which have been shown to have increased accuracy [29]. 

Where the current study falls short in terms of experimental animal numbers, it is able to show a clear trend of significant increase in CSF IL-6 alongside a WCC increase, and the WHO recommended staging determinant, as well as a strong association with astrocytosis in the brain. It is recommended that further characterization of the role of IL-6 in disease progression should be investigated and in a larger experimental group which would verify the current study's results and help to confirm the utility of IL-6 in stage determination.

## 5. Conclusions

The current study was conducted in a vervet monkey HAT model which has shorter periods to relapse as well as induced meningoencephalitis induction and which can allow for novel diagnostic marker testing and application. The study shows that IL-6 in CSF is elevated in late-stage infection of HAT and is accompanied by a rise in WCC, total protein and reactive astrogliosis. Thus, IL-6 has potential for use as an adjunct late-stage disease biomarker in the Rhodesian sleeping sickness.
